# Response to PD-1 inhibition in MMRd/MSS pancreatic ductal adenocarcinoma: the relevance of parallel testing

**DOI:** 10.1007/s00432-025-06334-3

**Published:** 2025-10-21

**Authors:** Heike L. Pahl, Silke Lassmann, Anne M. Schultheis, Stephan Rau, Melanie Börries, Justus Duyster, Matthias Zaiss, Michael Quante, Heiko Becker, MTB-FR Network

**Affiliations:** 1 Department of Medicine I , Hematology/Oncology and Stem Cell Transplantation, Medical Center – University of Freiburg, Faculty of Medicine, University of Freiburg, Freiburg, Baden-Württemberg Germany; 2 Institute for Clinical Pathology, Medical Center – University of Freiburg, Faculty of Medicine, University of Freiburg, Freiburg, Baden-Württemberg Germany; 3 Department of Diagnostic and Interventional Radiology, Medical Center – University of Freiburg, Faculty of Medicine, University of Freiburg, Freiburg, Germany; 4 Center for Personalized Medicine, Medical Center – University of Freiburg, Faculty of Medicine, University of Freiburg, Freiburg, Baden-Württemberg Germany; 5 Institute for Medical Bioinformatics and Systems Medicine, Medical Center University of Freiburg, Faculty of Medicine, University of Freiburg, Freiburg, Germany; 6 Praxis Für Interdisziplinäre Onkologie & Hämatologie, Wirthstr. 11C, 79110 Freiburg, Germany; 7 Department of Medicine II, Gastroenterology, Medical Center – University of Freiburg, Faculty of Medicine, University of Freiburg, Freiburg, Baden-Württemberg Germany

**Keywords:** personalized medicine, molecular tumorboard, pancreatic adenocarcinoma, DNA mismatch repair, microsatellite stability

## Abstract

While pancreatic ductal adenocarcinoma (PDAC) carries a poor prognosis, a small fraction of patients show high microsatellite instability (MSI-H) and may respond to immune checkpoint inhibition. An MSI-H genotype is usually associated with deficiencies in the DNA mismatch-repair mechanism (MMRd). However, discordances between the mismatch-repair status by immunohistochemistry and the microsatellite status by molecular analyses have been noted. To date it is not clear whether PDAC patients with mutations in mismatch repair genes, which result in loss of protein expression (MMRd), who nonetheless retain microsatellite stability (MSS), can profit from checkpoint inhibitor therapy. Here, we present the case of a PDAC patient, diagnosed as MMRd/MSS, who responded to checkpoint inhibitor therapy after failing two lines of chemotherapy. Our data suggest that both MMR and microsatellite status should be determined in PDAC patients and that MMRd status alone, even in an MSS phenotype, can constitute an indication for checkpoint inhibitor therapy.

**Supplementary Information:**

The online version contains supplementary material available at 10.1007/s00432-025-06334-3.

## Introduction

Pancreatic ductal adenocarcinoma (PDAC) carries a dismal prognosis, 5-year survival rates remain at 10%, even when patients with limited, localized disease are included. However, over 80% of PDAC patients present with an inoperable stage at diagnosis, reducing these rates even further. Across a variety of solid tumor entities, the use of checkpoint inhibitor therapy has shown efficacy, especially in those tumors displaying mismatch-repair-deficiency (MMRd) and high microsatellite-instability (MSI-H) (Marabelle et al. 2025). While this phenotype is found in only 1–2% of PDAC tumors (Luchini and Scarpa 2023), they may respond to checkpoint inhibition, although response rates are lower than for example in colorectal cancer with MSI-H (Taïeb et al. 2023; Chakrabarti et al. 2022). Identification of patients likely to benefit from this therapy is particularly important in this entity, given the aggressive disease course.

The terminology “MMRd/MSI-H” is frequently used interchangeably, implying that mismatch-repair-deficient tumors are always microsatellite-instable and vice versa. However, discrepancies between the cause (“MMRd”) and the predicted consequence (“MSI”) have already been noted in various entities. For example, in 666 cases of endometrial cancer, discordant results between the microsatellite (MS) status and the mismatch-repair (MMR) status were found in 3.8% (n = 25) of patients. In most cases, the discrepancy arose from clonal heterogeneity and the presence of subclones (Riedinger et al. 2024). Likewise, discrepancies between MS and MMR status have been reported in patients with sarcoma and endometrial cancer (Fernandes, et al. 2024; Mendiola et al. 2023). In a large cohort of 1306 patients, Nádorvári and colleagues found discrepancies between MMR and MS status in 19,3% of cases (Nádorvári et al. 2024), identifying preanalytical handling as the most important factor. Excluding technical challenges, in a seminal study of 585 cases, Jaffrelot et al*.* define unusual MMRd phenotypes in 15% (n = 89) of non-colorectal cancers, which frequently did not result in an MSI-H status (Jaffrelot et al. 2022).

MMR/MSI testing was initially developed to aid in the diagnosis of HNPCC (hereditary non-polyposis colon cancer). The genomic loci selected for PCR-based detection of MSI were therefore validated in these patients (“Bethesda” MSI PCR Panel 1 and 2). Subsequently, this PCR-based MSI detection strategy was broadly adopted to predict response to checkpoint-inhibition therapy in various entities. However, mutations in the four MMR proteins MLH1, MSH2, MSH6 and PMS2, may not necessarily cause alterations in either the 5 or the 10 MSI-loci represented in the HNPCC PCR-panels. Therefore, the College of American Pathologists (CAP) proposed guidelines for MMR and MSI testing in patients considered for immune checkpoint inhibitor therapy. The CAP guidelines were endorsed by the American Society of Oncology (ASCO) in 2022 (Vikas et al. 2023).

Importantly, while the ASCO-CAP guidelines give specific recommendations for colorectal cancer (CRC), for gastroesophageal and small bowel cancer as well as for endometrial cancer, no recommendation in favor of any testing method over another was made for other cancer types, due to lack of sufficient evidence. Nonetheless, the guidelines already address the occurrence of discordant results. In these cases, it is recommended “that pathologists should interpret any evidence of MMR deficiency by IHC or MSI by NGS or PCR as a positive result for patients to be eligible for immune checkpoint inhibitor therapy” (Vikas et al. 2023). However, very few clinical outcomes of patients with discrepant MMR/MSI status, treated with immune checkpoint inhibition have been reported outside the four cancer entities specifically mentioned in the ASCO-CAP guidelines. Here, we present a patient with PDAC, diagnosed as MMRd but MSS, who responded to checkpoint inhibitor therapy after failing two lines of chemotherapy.

## Results

### Case presentation

A 59-year-old man with a history of relapsed seminoma, treated surgically as well as with radiation therapy to the paraaortal and inguinal lymph nodes 22 years previously, was referred to our institution for the management of PDAC associated with a high-grade intraductal papillary mucinous neoplasm (IPMN). His family history was notable for multiple neoplastic diagnoses in first degree relatives, including his mother deceased from ovarian cancer at the age of 54. The initial staging showed irresectable disease, as the laparoscopic exploration revealed deep venous infiltration as well as a direct contact of the tumor to the superior mesenteric artery. An osteolytic lesion in lumbar vertebral body1 (LWB1) was suspicious of metastasis, but was not histologically confirmed as this did not impact the therapeutic decision.

**Fig. 1:**
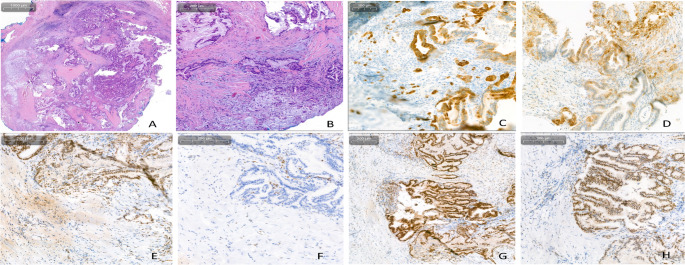
MMR and PD-L1 protein expression in the PDAC lesion. **A** and ** B** Hematoxylin and Eosin (HE)-staining demonstrating (**A**) the IPMN- and (**B**) the invasive component of the lesion. **C** Immunohistochemical staining for CK7. Strong CK7-expression in single cells and small cell clusters marks the invasive component invading the stroma. **D** PD-L1 staining (**E–H**) Immunohistochemical expression of the four MMR proteins: **E** MSH2, **F** MSH6, **G** MLH1, **H** PMS2

The patient received four courses of FOLFIRINOX therapy, following which, due to progressive disease, a second line treatment with gemcitabine and nab-paclitaxel was initiated. Immunohistochemical staining of the diagnostic biopsy had shown a MMRd phenotype with a loss of MSH6 protein expression both in the IPMN component and in the carcinoma cells (Fig. 1). However, subsequent analysis of 8 microsatellite loci by PCR revealed a discrepant MSS status (Supplemental Fig. 1). Discordance between microsatellite status and MMR immunophenotype has been previously described in several cancer entities, but is exceedingly rare in PDAC (Jaffrelot et al. 2022).

Because of this discrepancy the patient was presented to the Molecular Tumorboard, which recommended extended molecular diagnostics including sequencing of a TSO gene panel. Concordant with the lack of MSH6 protein in immunohistochemical stainings, DNA extracted from the paraffin embedded tumor lesion carried a mutation in MSH6 (p.X1267_splice), classified as pathogenic and likely pathogenic in the InSiGHT and ClinVar Databases, respectively. The lesion also carried a concomitant mutation in the exonuclease domain of *POLD1* (p.T473M), classified as likely benign by the REVEL algorithm and shown not to confer the “ultramutated” phenotype characteristic of POLD1 mutations (Ambrosini et al. 2024), as well as a *KRAS* (G12V) variant. The sections used for DNA extraction contained approximately 20% tumor cells. Based on their variant allele frequencies (VAF), 10.3%, 8% and 8,5%, respectively, all three alterations were acquired somatically, and, most likely, are present in all tumor cells. Importantly, only 2.48% of the 121 microsatellite loci represented in the NGS-panel were unstable, confirming the MSS status. The tumor mutational burden (TMB) was only slightly elevated, at 12,6 mutations per megabase. In addition, immunohistochemical PD-L1 staining revealed the following scores: TPS 10%, CPS 35, IC-Score 3 (Table 1).

**Table 1:** Molecular and genetic changes found in the presented patient

	Mutations		
Gene/locus	Protein alteration	Classification	VAF (Variant Allele Frequency) 20% tumor cells in biopsy
KRAS	p.G12V	Pathogenic^1^	8.5%
TP53	p.R248Q	Pathogenic^1^	8.5%
MSH6	p.X1267_splice	Likely pathogenic^1^/pathogenic^2^	10.3%
POLD1	p.T473M	Unclear significance^1^/likely benign^3^/does not confer POLD1 signature (Ambrosini et al. 2024)	8%

Other molecular findings		
Analysis	Results	Method
Microsatellite stability	MSS 2,48% instable loci (121 loci)	NGS, TSO 500
Microsatellite stability	MSS 0 instable loci (0/8)	PCR
MMR status	MMRd loss of MSH6 staining	IHC
PD-L1 staining	TPS 10%, CPS 35; IC-Score 3	IHC
TMB	12.6 mutations /Mb	NGS

IHC: immunohistochemistry; NGS: next generation sequencing; PCR: polymerase chain reaction; TMB: tumor mutational burden. 1: ClinVar (Landrum et al. 2016); 2: InSIGHT (Plazzer et al. 2013); 3: REVEL (Ioannidis et al. 2016)

Despite the MSS genotype, this comprehensive molecular profiling thus provided several rationales for the use of checkpoint inhibition therapy:

1. A MMRd status by immunohistochemistry
2. A likely pathogenic MSH6 variant by sequencing
3. Elevated PD-L1 expression

**Fig. 2:**
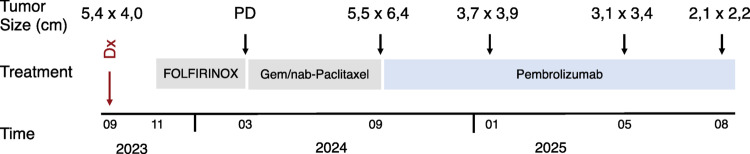
Treatment Course. Upon diagnosis of PDAC, initial molecular pathological analyses were performed. The patient received first line treatment with four cycles of mFOLFIRINOX and, upon progression, second line therapy with gemcitabine and nab-paclitaxel. During this time, he was presented to the molecular tumorboard and additional molecular analyses were performed. When staging after 4 cycles of gemcitabine/nab-paclitaxel again showed progressive disease, following recommendation of the molecular tumorboard, treatment with pembrolizumab was initiated in 09/2024

**Fig. 3:**
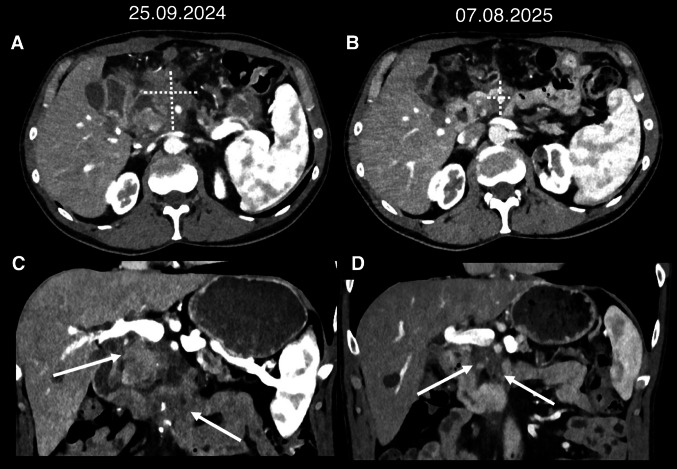
Arterial phase images of photon-counting CT scans before the initiation of pembrolizumab (**A**, **C**) and after 11 months of treatment (**B**, **D**). (Top) Axial plane reconstructions with measurements: **A** 5.5 × 6.4 cm, **B** 2.1 × 2.2 cm. (Bottom) coronal plane reconstructions: arrows point to the tumor in the pancreatic head, which can be distinguished from the pancreatic parenchyma due to its hypodense appearance

Following rapid progression under second line chemotherapy, with de novo manifestation of peritoneal carcinosis, the patient initiated pembrolizumab treatment in 09/2024. Staging following 3, 6 and 11 months of anti-PD-1 immunotherapy each showed a partial response, accompanied by a continuous substantial reduction in tumor volume, now measuring 2,1 × 2,2 cm while it was denoted at 5,5 × 6,4 cm before the initiation of checkpoint inhibitor treatment (Figs. 2 and 3). Moreover, dilation of the pancreatic duct was reduced as was the extent of peritoneal carcinosis. The patient is currently continuing pembrolizumab monotherapy and enjoying a good quality of life, able to work part time running his own business.

## Discussion

Given the possible therapeutic impact of immune checkpoint inhibition, identifying patients likely to profit from this therapy is essential. Response to immune checkpoint inhibition is highest in cancers often summarily identified as “mismatch-repair-deficient” and “microsatellite-instability-high” (MMRd/MSI-H)”. This implies that the two phenotypes, MMRd and MSI-H are superimposable. While MMR deficiency does frequently lead to microsatellite instability, the concordance of the two phenotypes is variable among different cancer entities (Riedinger et al. 2024; Fernandes et al. 2024; Mendiola et al. 2023; Nádorvári et al. 2024).

Technical aspects may account for some of the variability observed (Nádorvári et al. 2024). While MMR status is uniformly determined by immunohistochemical (IHC) staining for MLH1, PMS2, MSH2 and MSH6, microsatellite stability is determined by polymerase chain reaction (PCR) or increasingly by next generation sequencing (NGS). Recently, recommendations for testing have been proposed that specify quality control measures, including DNA quality and assay sensitivity, as well as a tumor cell content of at least 15–20% (Vikas et al. 2023). This should minimize some of the variability previously observed, which was attributed to technical aspects (Nádorvári et al. 2024). More importantly, biological properties of the tumor, for example the presence of subclones, may account for discrepant results (Riedinger et al. 2024). However, it has recently become clear that the pathophysiology is more complex and accounts for the variable proportion of MMR/MSS discordant tumors across the different entities.

In a seminal study, Jaffrelot and colleagues analyzed 585 non-colorectal MMRd tumors, defining “classical MMRd” as a loss of IHC staining for both partners of the MMR protein heterodimers (MLH1/PMS2 or MSH2/MSH6) with a concomitant MSI-H phenotype. Tumors not meeting these two criteria were termed “unusual MMRd phenotypes”, present in 15% (n = 89) of the samples. They classified these into four subgroups: (i) isolated loss of PMS2 or MSH6, (ii) classical loss of MLH1/PMS2 or MSH2/MSH6 without MSI, (iii) four MMR proteins retained with MSI and, (iv) complex loss of MMR proteins. A large proportion of the unusual MMRd phenotypes showed an MSS or MSI-low phenotype. As only five of the 89 patients were treated with immune checkpoint inhibition, the authors were not able to draw any conclusions on the impact of the unusual MMRd phenotypes or the MSS status on therapeutic response.

Our patient falls into category i) defined by Jaffrelot et al., isolated loss of MSH6 protein expression with retention of microsatellite stability (MSS) by PCR and NGS testing. It has been previously shown that MSH3 can partially substitute for the loss of MSH6 function, providing a mechanistic explanation for this seeming discordance (Umar et al. 1998). Consequently, a more subtle manifestation of MSI-H with only minor and discrete shifts by MSI-PCR has been described in tumors with isolated loss of MSH6 (Stelloo et al. 2017). Despite the MSS status, our patient responded well to pembrolizumab, after having failed two previous chemotherapy regimen.

This case thus argues strongly for a comprehensive biomarker profiling in patients with KRAS mutant PDAC, that includes determination of both MMR and MS status independently for each patient. In centers where NGS is not routinely used, MMR status may be determined by IHC, while MS status is assessed by PCR. If NGS is used, it will need to be determined whether detection of a mutation in one of the four MMR genes is sufficient to define an MMRd phenotype or whether confirmation of loss of protein expression is required, at least for previously unknown alterations. Irrespective of the method used, either an MMRd or an MSI-H phenotype should prompt consideration of immune checkpoint inhibitor therapy.

## Methods

### Patient consent

The patient provided written consent, in German, to have his data published in an anonymized form.

### DNA based methods

The biopsy of an IPMN lesion with associated ductal carcinoma was analyzed for MS status by PCR and capillary electrophoresis (LMR MSI Analysis Kit, Promega) as well as for MS status, tumor mutational burden and multiple gene alterations by NGS panel sequencing (TruSight Oncology 500 panel, Illumina) in an accredited molecular pathology diagnostic laboratory. NGS coverage was 570x.

### Immunohistochemistry

Immunohistochemical staining for MLH1 (ES05; Agilent Technologies, Inc, Santa Clara, USA), PMS2 (EP51; Agilent Technologies, Inc, Santa Clara, USA), MSH2 (FE11; Agilent Technologies, Inc, Santa Clara, USA) and MSH6 (EP49; Agilent Technologies, Inc, Santa Clara, USA, which is directed against a synthetic peptide at the N-terminal region of the protein) as well as PD-L1 (SP263; Ventana®) was performed according to manufacturer´s protocol and evaluated by a board certified pathologist in an accredited pathology department according to current evaluation criteria (Bartley et al. 2022).

## Supplementary Information

Below is the link to the electronic supplementary material.

Supplementary file1 (DOCX 589 KB)

## Data Availability

No datasets were generated or analysed during the current study.
