# Supplementary material for: Response to PD-1 inhibition in MMRd/MSS pancreatic ductal adenocarcinoma: the relevance of parallel testing
Source: J Cancer Res Clin Oncol. 2025 Oct 21;151(12):302. doi: 10.1007/s00432-025-06334-3 (PMC12540230; doi:10.1007/s00432-025-06334-3)
Supplement: Supplementary file 1 — Supplementary file1 (DOCX 589 KB) [file 432_2025_6334_MOESM1_ESM.docx]

**Supplemental Figure 1: MSI testing by PCR and capillary electrophoresis.**  **(Top)** Electropherograms MSI PCRs of normal controls and patient tumor sample (8 loci separated by color-labelling and size distribution) **(Bottom)** positive and negative technical controls.
